# Hybrid Systems to Boost EEG-Based Real-Time Action Decoding in Car Driving Scenarios

**DOI:** 10.3389/fnrgo.2021.784827

**Published:** 2021-11-29

**Authors:** Giovanni Vecchiato

**Affiliations:** Institute of Neuroscience, National Research Council of Italy, Parma, Italy

**Keywords:** hybrid systems, action prediction, driving, EEG, EMG, EOG

## Abstract

The complexity of concurrent cerebral processes underlying driving makes such human behavior one of the most studied real-world activities in neuroergonomics. Several attempts have been made to decode, both offline and online, cerebral activity during car driving with the ultimate goal to develop brain-based systems for assistive devices. Electroencephalography (EEG) is the cornerstone of these studies providing the highest temporal resolution to track those cerebral processes underlying overt behavior. Particularly when investigating real-world scenarios as driving, EEG is constrained by factors such as robustness, comfortability, and high data variability affecting the decoding performance. Hence, additional peripheral signals can be combined with EEG for increasing replicability and the overall performance of the brain-based action decoder. In this regard, hybrid systems have been proposed for the detection of braking and steering actions in driving scenarios to improve the predictive power of the single neurophysiological measurement. These recent results represent a proof of concept of the level of technological maturity. They may pave the way for increasing the predictive power of peripheral signals, such as electroculogram (EOG) and electromyography (EMG), collected in real-world scenarios when informed by EEG measurements, even if collected only offline in standard laboratory settings. The promising usability of such hybrid systems should be further investigated in other domains of neuroergonomics.

## Introduction

Today's human at work is asked to continuously interact with objects and the environment to perform a wide variety of tasks. In this regard, the research field of neuroergonomics aims to unravel the neural bases of those neurophysiological processes involved in the interaction between the user and a technical system during everyday life activities (Parasuraman, 2003; Dehais et al., 2020; Gramann et al., 2021).

Because of its complexity, one of the main real-world activities targeted by neuroergonomics is driving (Navarro et al., 2018). Studies demonstrated that driving behavior is the final result of simultaneous mental processes such as attention, decision-making, vigilance, motor, and cognitive control (Calhoun et al., 2002; Calhoun and Pearlson, 2012). Driving activity can cause drowsiness, fatigue, and an increase in workload, and it is one of the primary causes of death worldwide (Borghini et al., 2014). In order to assure road safety, it becomes fundamental to have a deep understanding of those mental processes underlying the interactions existing among the driver, the car, and the external environment to predict human behavior resulting in steering and braking actions. In recent years, driving scenarios have been enriched by technological advancements in designing autonomous cars (Badue et al., 2021). However, even if intelligent systems can execute actions on behalf of the driver, the correctness of these choices can only be evaluated once we understand those mechanisms underlying the driver's behavior in simulated and real traffic scenarios. In this regard, expertise could be an essential factor worth considering since evidence collected among professional and non-expert drivers suggest that the two populations share basic neurophysiological mechanisms, whereas the expertise subtending exceptional driving abilities may be associated with specific morphological and functional cerebral architecture changes (Bernardi et al., 2013, 2014).

For this reason, several pieces of research have been conducted to identify the neural basis of transportation and car driving. A recent meta-analysis presents a neuroergonomic framework according to which the neural bases of driving behavior are categorized into strategical (i.e., navigation), tactical (i.e., overtaking), and operational (steering and braking) tasks (Navarro et al., 2018).

In this context, developing efficient brain-based systems for the real-time decoding of brain processes underlying driver's behavior would be highly beneficial for the design of assistive devices.

This perspective provides a succinct overview of the literature about hybrid systems used for action prediction and the related limitations. Then, it presents the results related to car driving scenarios as proof of the level of technological maturity achieved in the last years. In this context, driving actions are predicted (i) exploiting secondary tasks eliciting cerebral activity related to a higher level of motor control and (ii) by measuring neural correlates of motor preparation as a marker of braking and steering actions and. This methodological approach could benefit additional ecological scenarios in neuroergonomics, such as telerehabilitation and occupational safety.

## From EEG-Based Action Decoders to Hybrid Systems

Electroencephalography (EEG) is one of the most used techniques for monitoring brain signals in operational environments. This measure provides the variation of electrical potentials on the scalp surface, generated by the summation of post-synaptic potentials within cortical layers (Biasiucci et al., 2019). Electroencephalography has the critical advantage of tracking brain dynamics with millisecond accuracy and is used in real-world scenarios for neuroimaging studies outside the lab. The evolution of technology allowed the removal of wires and produced wearable and long-lasting recording devices, enabling a wide range of experiments in real-world settings (Debener et al., 2012; Mihajlović et al., 2015; Mullen et al., 2015; Casson, 2019).

The integration of EEG-based action predictions into the control of an assistive technology device, such as a car, would have the great advantage of detecting, as early as possible, the movement preparation and execution, both in a laboratory and in more natural environmental settings. However, despite the noteworthy technological advancement of the last decade, there are still several issues that limit the utilization of the EEG for the real-time monitoring of actions in working environments. For instance, there is the need to improve the EEG hardware to obtain recordings more robust to artifacts and longer battery life and produce smaller devices to be socially accepted by everyone. Other psychophysiological and technological constraints make this prediction hard to achieve in real-life scenarios. Factors such as attention, memory load, fatigue, and competing cognitive processes (Gonçalves et al., 2006; Käthner et al., 2014; Calhoun and Adali, 2016), as well as user's individual characteristics such as lifestyle, gender, and age (Kasahara et al., 2015) influence brain dynamics producing significant intra- and inter-subject variability (Saha and Baumert, 2020; Saha et al., 2021). Common EEG artifacts generated by muscles and eye movements, impedance shifts, environmental noise are typically amplified in real-world scenarios, sensibly affecting the quality of EEG signals during real-time monitoring (Waard, 1996; Zander et al., 2017; Lohani et al., 2019). Also, wearing an EEG device for users within operational environments could be uncomfortable and lead to the corruption of the underlying brain processes. Although the technology provides researchers with high-impedance systems equipped with active shielded electrodes for mobile applications, these devices do not solve all the mentioned issues intrinsically characterizing all ecological environments. This low signal-to-noise ratio returned by raw EEG data requires the use of a range of conceptually very different and computationally expensive algorithms to extract significant temporal and frequency EEG features (Müller et al., 2004; Lotte et al., 2007, 2018; Krusienski et al., 2011; Bellotti et al., 2019). These algorithms often are demanding in terms of calibration because requiring large training sets and are not robust to real-life environmental noise affecting EEG recordings. Other issues relate to the high-dimensionality and non-stationarity of the EEG data, impacting the classification performance (Lotte et al., 2018). In addition, most of the classification methods used in the literature are applied for offline EEG analyses, thus requiring the improvement of this methodology for online applications to guarantee a computational efficiency for the real-time decoding of the brain activity. Hence, the computing hardware and software must warrant a sufficiently high performance and low latency to preserve the earliness of prediction (Wöhrle et al., 2017).

Hence, different physiological, behavioral, and technical data can be combined to improve the reliability of EEG-based predictions and their fully automated application for supporting the user in self-paced movements in critical environments. For example, the prediction of actions onset based on EEG analysis can be improved by the design of hybrid systems simultaneously monitoring additional peripheral signals, such as electroculogram (EOG) and electromyographic (EMG) data, depending on the context requirements (Kirchner et al., 2014). The hybrid concept was introduced in the field of the Brain Computer Interfaces (BCIs), exploiting advantages of different physiological signals and computational approaches to finally achieve specific goals better than a conventional EEG based system, such as improving the overall classification rate or reducing the rate of false positives (Pfurtscheller et al., 2010; Li et al., 2019). Hybrid systems should rely at least on one brain signal in the form of electrical, magnetic, or hemodynamic changes, and at the same time, they can incorporate peripheral or external signals to improve the whole system's performance. For instance, combinations of eye movement signals with neuronal signals usually are utilized for hybrid EEG–EOG BCIs (Usakli et al., 2009, 2010; Ma et al., 2015; Hong and Khan, 2017). Hence, the design of hybrid systems can improve the action prediction performance depending on the particular application.

Electroencephalography and EMG signals can be used to predict movements before the action onset reliably, showing that multimodal machine learning approaches can be potentially used to control an electronic device (Kirchner et al., 2014; Wöhrle et al., 2017). Unimodal EEG-based predictions can be achieved earlier with respect to unimodal EMG-based prediction, thus suggesting that EEG is more suitable for providing the user the feeling that a device delivers support on time without significant delay. Also, EEG analysis leads to more false positives than EMG due to the higher signal-to-noise ratio characterizing such neural data. In addition, which signals are relevant at which state of movement planning and execution have been systematically investigated with machine learning approaches to predict movement targets (Novak et al., 2013). This study reports that each sensing modality has its peculiarities. Electroencephalography is suitable for very early prediction or if the user cannot perform the movement. Electromyography and hand position are accurate after limb motion onset. Eye-tracking is accurate at motion onset, but it is not able to predict motion dynamics. Combining EEG and EOG results in higher accuracy than using a unimodal approach and is convenient since the two signals are often measured together. Augmenting EMG with eye-tracking allows predictions to be made earlier than with only EMG. However, this research field is not mature yet to make precise comparisons of performance and calibration times between machine learning approaches for unimodal and multimodal measurements.

Several challenges also characterize these hybrid systems. One of the significant issues in this research is identifying the best combinations of signals to reach the best prediction performance since the optimal combination could differ across users and experimental scenarios. Variables including system complexity, cost, user workload have to be evaluated when comparing hybrid systems with unimodal predictions. From the user's point of view, the complexity of hybrid systems is usually higher than that of conventional single modality recordings because they are required to wear multiple brain and body sensors. User acceptability is a crucial criterion that needs to be considered in designing and implementing such systems (Pfurtscheller et al., 2010; Li et al., 2019).

## Hybrid Systems in Car Driving Scenarios

In the field of driving research, several studies addressed the issue of action detection and prediction based on the discrimination of different EEG features in simulated (Haufe et al., 2011; Gheorghe et al., 2013; Khaliliardali et al., 2015; Kim et al., 2015; Vecchiato et al., 2018, 2020, 2021) and real driving scenarios (Haufe et al., 2014; Zhang et al., 2015).

A few studies used secondary tasks to elicit neural features predicting steering during simulated and real car driving. In particular, the contingent negative variation (CNV) potential was generated by a go/no-go task to investigate the decoding of drive and brake events (Gheorghe et al., 2013; Khaliliardali et al., 2015). Results suggested that these actions can be discriminated around 320 ms before the movement with a classification performance of 0.77. In addition, Zhang et al. (2015) described an online event-related negativity (ERN) classifier to predict steering events, guided by a directional cue, both in a laboratory and real car driving scenarios. In both experimental conditions, they discriminated correct by error trials 480 and 700 ms after the directional cue. The classification performance is 0.70, but the computational timing cost is not reported, so the time interval between the directional cue and the classifier decision is unknown, and therefore whether it comes before or after the actual movement execution.

Other studies investigated the driver's action without using external cues with the advantage of limiting the additional driver's mental load. Haufe et al. (2011) explored pseudo-online emergency braking detection and evaluated that such a system in a simulation environment could eventually detect foot action around 130 ms before its onset. The possibility to decode self-generated actions detecting steering was also assessed, and in particular, whether the driver would perform a lane change in a simulated highway was predicted about 800 ms earlier the action onset with a true positive rate of 74.6% (Gheorghe et al., 2013). In addition, Vecchiato et al. (2018) identified an EEG independent component associated with the fronto-central electrodes exhibiting synchronization of theta EEG rhythm around 800 ms before the braking onset.

In line with the concept of hybrid systems, Kim et al. (2015) proposed a combination of EEG features in the time and frequency domains to distinguish three different kinds of stimulus-driven brake situations (i.e., sharp, soft, no brake). This study reported the highest EEG response-locked decoding performance at −480 and −420 ms distinguishing sharp and soft braking from no braking, respectively. It was harder to classify sharp and soft braking conditions with the same method (largest difference at −160 ms), which returned lower performance than a classifier based on EMG features. There were also significant results with hybrid decoding systems in a real car scenario where participants were asked to drive on a non-public test track (Haufe et al., 2014). They reported that a hybrid (EEG and behavioral features) classifier detected emergency braking even earlier than the laboratory setting (around 300 ms before the braking onset) (Haufe et al., 2011).

Moreover, in order to characterize the relative contribution of the EEG associated with the preparation of natural and self-initiated steering actions while driving to investigate its predictive power, the EEG related to *continuous steering* during the driving simulation was tested by means of canonical correlation analysis (CCA) and a linear lagged regression approach (LLR) to identify the relative contribution of the EEG signals in steering anticipation (Di Liberto, accepted). Results showed that the combination of CCA-LLR analysis is valuable to disentangle the relative contribution of behavioral and electrophysiological components—within the EEG signals—for steering prediction in a continuous driving simulation task. This result demonstrates that brain-related EEG signals significantly improve the overall decoding performance, showing that the significant contribution in predicting steering comes from non-brain-related signals, such as ocular and muscular components.

**Figure 1:**
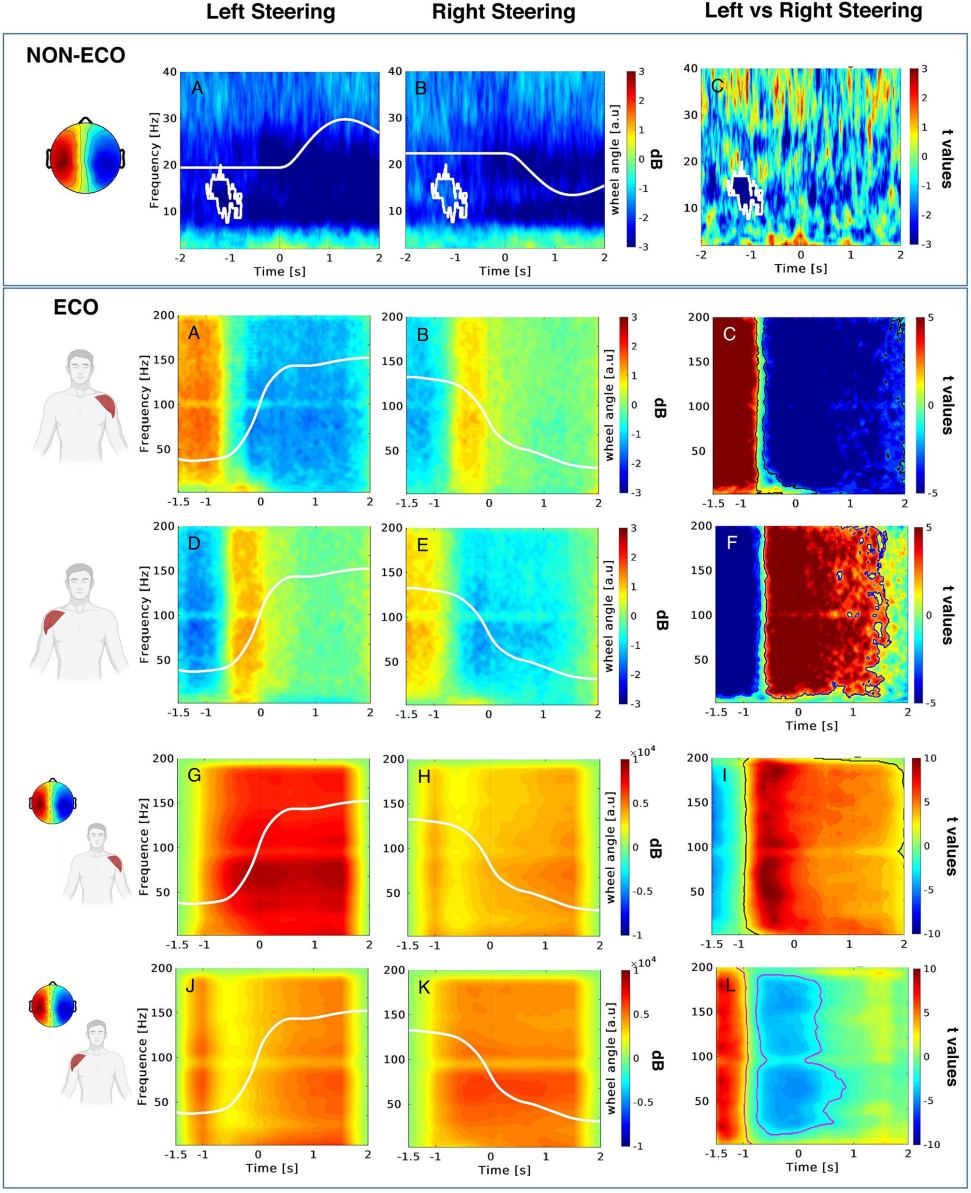
**NON-ECO frame**. Time-frequency EEG patterns collected during left **(A)** and right **(B)** non-ecological steering, as well as their statistical comparison **(C)**. The topography in the left part of the picture shows the average scalp map related to the cluster of independent components. **ECO frame**. ERSP for the EMG signals collected during the non-ecological steering task **(A–F)**, and cross-correlation results between EEG and EMG data **(G–L)**. The first (second) row (from the top) illustrates the EMG ERSP for the left (right) deltoid during left and right steering, as well as their statistical comparison. The third (fourth) row illustrates the EEG-EMG cross-correlation values for the left (right) deltoid during left and right steering, and the statistical comparison of the two conditions. White lines depict the left and right steering wheel angle profiles. Color bars indicate in blue (red) the decrease (increase) of EEG, EMG, and cross-correlation, as well as the statistical differences corresponding to the decrease (increase) of such activity during the left (right) steering. White and black masks delimit the statistically significant portion of the EEG, EMG, cross-correlation panels (adapted from Vecchiato et al., 2021).

Brain and muscular activities underlying steering behavior were also investigated with the final aim to increase the overall ecology of the experimental setting (Vecchiato et al., 2021). In particular, EEG feature predicting steering action and direction elicited by responding to traffic signs displayed on a computer screen was extracted and later exploited to increase the predictive power of the EMG collected in a more ecological steering task, such as a driving simulation. The desynchronization of the mu rhythm during the motor preparation of non-ecological steering cued by the traffic sign discriminated the muscular activity of the deltoids, thus anticipating subject steering behavior of 1.5 s. In addition, the increase of EMG activity of the deltoids anticipated the contralateral steering in both non-ecological and ecological steering tasks of 200 and 500 ms relative to the action onset, making it possible to discriminate such a driving behavior. Although these variations of EMG activity appear before the action onset allowing for possible online predictions, EEG data were used to increase the available time to perform such a calculation. The identified *non-ecological EEG* feature correlated with the *ecological EMG* activity of the deltoids, providing an improvement of the discrimination power of the steering side during driving simulation (Figure 1). These findings show an approach to increase the ecology of the experimental setting by limiting the invasiveness of the neurophysiological measurements using surface EMG sensors in the ecological scenario and combining neural data collected in the non-ecological one. This approach provides a way to monitor the user performance online through a simpler to acquire muscular correlate when compared to neural data, which could be recorded offline to increase the decoding system's performance without impacting the complexity of the ecological setting.

## Conclusions

The coupling between EEG, EMG, and ocular signals is a valid mechanism for utilizing hybrid systems for the detection and online prediction of driving actions, exemplifying how it might be possible to complement information from behavioral, physiological, and external sources to control the level of assistance needed by the driver in that context (Chavarriaga et al., 2018). This methodology could pave the way for the utilization of hybrid systems based on neural signals—collected in standard laboratory settings and processed offline—having the role in improving the predictive power of peripheral signals—collected in more ecological settings and possibly processed online—correlated with the upcoming action execution.

The predictive power returned by coupling the EEG with peripheral signals demonstrated in car driving scenarios could be further investigated in larger sets of actions to extend the validity of this approach to other neuroergonomic areas. For instance, this methodology could foster the spread of mobile brain and body applications (Makeig et al., 2009; Gramann et al., 2011) and BCI paradigms (Douibi et al., 2021; Saha et al., 2021) onto several other contexts of our daily life. The capability to remotely monitor in an ecological way an individual's action would have a tremendous impact in the rehabilitation field (Nuara et al., 2021), with the possibility to verify the compliance and adherence to treatment relieving the patient and caregivers from a massive burden in terms of time and costs. Applications could also extend beyond the clinical realm, virtually to any fields where action surveillance would be valuable for preventing harmful consequences. It is the case, for instance, of the occupational safety of workers dealing in their routine with unsafe practices, for whom the use of this ecological methodology could reduce the likelihood of occupational injuries during the performance of high-risk motor tasks (Rizzolatti et al., 2021).

## Data Availability Statement

The original contributions presented in the study are included in the article/supplementary material, further inquiries can be directed to the corresponding author/s.

## Author Contributions

The author confirms being the sole contributor of this work and has approved it for publication.

## Funding

This study received funding from CAMLIN Limited. The funder was not involved in the writing of this article or the decision to submit it for publication.

## Conflict of Interest

The author declares that the research was conducted in the absence of any commercial or financial relationships that could be construed as a potential conflict of interest.

## Publisher's Note

All claims expressed in this article are solely those of the authors and do not necessarily represent those of their affiliated organizations, or those of the publisher, the editors and the reviewers. Any product that may be evaluated in this article, or claim that may be made by its manufacturer, is not guaranteed or endorsed by the publisher.
